# Our Poorer Neighbours

**Published:** 1889-08-10

**Authors:** 


### Our Poorer Neighbours.

### HOW TO PROVIDE FOR THE WINTER'S DISTRESS.

Last Saturday afternoon a conference of workers was held in All Saints' Schoolroom, Kensington-park-road, to consider the question of "Help for our Poorer Neighbours, with a View to next Winter's Needs."

Canon Trench, vicar of the parish, presided, and said he had sent notice of the present meeting to the various religious bodies round about there, as well as to Church clergymen, and to the local representatives of the Charity Organisation Society, as they sought the judgment upon the question before them of all engaged in charitable relief. Visiting the poor and helping them would never, he supposed, become a really popular occupation, for if continuously undertaken it was laborious, and called for a good deal of self-denial. Occasionally, however, it was even fashionable, and during the last twenty years, at all events, real progress had been made in the direction to be desired. What they wanted now were scientific methods for doing this kind of work, as well as for every other kind. People, full of kindness to the poor, were often disappointed with the unfortunate result of their good intentions. He thought it a real kindness towards these people to place at their disposal the experience of those much engaged in this work, and able to advise as to the best methods tending to the best results. They had the advantage now of the presence of Mr. Henry C. Burdett, well known to them all, and known to some of them, at all events, as one who took a prominent interest in philanthropic work, throwing into it the same skill and energy that he employed in everything he undertook.

Mr. Henry C. Burdett said there had also been a second question set down for discussion, "Our Sick Poor: How we can best help them," but he would limit himself to the one question, "Help for our Poor Neighbours, with a View to next Winter's Needs." This meeting was held now, because much of the distress, and much of the unintelligent and helpless way they sought to grapple with it, was due to leaving many things to the last moment which ought to be in order when required. He presumed he was addressing the active workers and thinkers of this district; any others would hardly attend a meeting of this kind on a summer afternoon. If they had good workers and officers for the commencement of any new departure, this was as much as was necessary or desirable. Those present, however, were Churchmen and Churchwomen of All Saints' congregation, parish, and district, and he thought if they were really to do the best for the whole district, this conference must be followed by a second, composed of all their neighbours who were working in any sense amongst the poor. The work was one not confined to any particular body of men or women, or any class, or any sect, or any school. Helping the poor was a duty devolving upon every thinking man and woman, and to secure adequate results they required united action from all. They required a method by which they could be assured concerning all distress, whether exceptionally great or simply acute, or chronic, that no person in their district could starve without their knowing of it, without their having the ability to help, and without their being able to deal with the case promptly and in the best way. What applied to one district in a town like London would apply to all districts, and the reason why London appeared to many to be helpless as well as hopeless was because of the vastness of the population. Many a noble-hearted man held back, fearful to come forward, because of the enormous mass of misery and the enormous population.

The plan he had to submit was not original. It had been worked with success in Boston, in the United States, where every person's need was known, and dealt with in a thorough and complete way. They would remember the Mansion House Fund of some four years ago, raised and spent when there was a great outcry of distress in London. He ventured to say, not without adequate knowledge, it would probably have been better for the people if that fund had never been raised and distributed. Not that he belonged to any special group of men with a "hobby." He thought there was merit attaching to all systems, and though sympathising to a certain extent with the Charity Organisation Society, he had never joined it. He had used it; and was sorry that society had let it be thought—he did not think it, but others did—that they were opposed to all prompt and adequate relief of all kinds under all circumstances. It would never do for them to attempt to deal with this question on any narrow ground, or as crotchet-mongers, or hobby-riders, but rather with large hearts and great intelligence. In London the residents had not the same touch with and knowledge of their poorer brethren which existed in provincial towns and small communities. Generally the obstacles in relieving the distress around them were the lack of all skill and lack of all hope. They had partially tried in Paddington the plan he desired to submit, and so great was their success, that last winter the poor there were able to do without any extraordinary help at all. Volunteer visiting was the only hope of civilisation against the gathering course of pauperism in great cities. Donations to existing charitable institutions might only be a means of shirking their plain duty, which was to face the difficulties presented by constantly-recurring distress in their own district, and see if it could not be effectively and permanently treated on some plan which offered hope of permanent alleviation. If they gave loans, they should be sure there were reasonable chances of repayment, else a loan encouraged a breach of faith; if a gift, it should be enough to put a family on a permanent footing of self-support. Small and uncertain doles were ruinous to the recipients. As to pensions in chronic cases, it should be understood that it would be reduced as the children became of working age, and, when the earnings were large enough, withdrawn altogether. Providing workmen with tools and clothes, and also the matter of emigration, were other questions which had to be considered. He touched upon these various points in order to show the need of adequate and accurate knowledge of the nature and extent of the distress to be relieved. In this district, accordingly, their first need was to obtain precise and accurate information:

I. Of all the relief given by the poor-law authorities and the various charities—that is, that a clearing-house of all the relief given shall be opened at once.

II. Of all the men and women who are out of work and who desire to obtain it.

The clergy would know that, by skilful management, sometimes whole families lived on charity alone. One went to church, another to chapel, a third to some other place of worship, a fourth interested some charitable agency, a fifth enjoyed the bounty of some lady at street-crossings, or otherwise. He was not sure he could not manage to get about £2 a week out of charity alone in London. (A laugh.) Recently, a man, with three children, was met by a clergyman who knew him, and said, "Well, Tom, where are you going?" "Going to beg." The clergyman told him the three children were of the same size, and the man took them back to where he got them, and started off with three children of the right ages (a laugh). [Instances were then given to show that people were often out of work because they had no desire to obtain it.] He would propose to open an office, to which all societies and individuals should be invited to send regular statements of the persons helped by them, with the character and amount of aid given in each case. A report could be returned every month to each society, showing which of its beneficiaries were assisted by others, and in what way. He could supply details, if this plan were favourably entertained. Alms were not the whole of charity, which must do four things: (1) Relieve worthy need promptly, fittingly, and tenderly; (2) prevent unwise alms to the unworthy; (3) raise into independence every needy person, where this was possible; (4) make sure that no children grow up to be paupers. He hoped the present movement might ultimately result in the establishment of an *association of friendly workers* to deal, on a lasting basis, with the distress in this district. This association should aim at these results: (1) thorough organisation; (2) bringing all workers amongst the poor into co-operation; (3) studying the whole problem and all its needs; (4) asking all the money needed for perfect machinery, and for sufficient relief; (5) asking a multitude of good men and women to help in the work; (6) asking of a few men and women a supreme devotion; (7) making sure that in every varying need and phase of distress, wise, prompt, and effective measures are taken to ensure the best possible remedy; (8) especially making sure that no children grow up paupers. He had always found it a mistake to ask for money before they had organised. They must set before people what they proposed to do, undertaking to do it as men of honour, and position, and knowledge, and saying "all we require is so much money." The organisation he proposed would consist of (1) a conference, (2) as many friendly visitors, men and women, being residents in the district, as might be attracted to the work, and the opportunities it afforded for readily helping and knowing the poor in their immediate neighbourhoods, (3) of a trained and, possibly, a paid agent or agents. He would gladly have his proposals printed so that workers might consider it in detail.

The Vicar said Mr. Burdett proposed a new departure, and the opinions of those present were invited.

Mr. Crispin (St. James's, Norlands) said he should be glad to have a printed copy of Mr. Burdett's plan, and could scarcely discuss its details till then. His experience about loans was that though some instalments might be paid, if was likely the whole would not be given back. As to the ignorance of the people, they were hoping that technical schools might do much good service. He thought the encouragement of thrift might bring hope to the poor, and it was especially desirable to dissuade them from early marriages. The want of common prudence in these matters was the explanation of a great deal of the poverty. The committee to which he belonged gave no assistance to people who were not known, who had not been in the parish for twelve months. The poor would be attracted to come and settle in any parish where it was an easy matter to obtain relief. Indiscriminate charity they discouraged. Where there was illness, and the children unable to provide for the wants of the family, they gave relief (small amounts—they could not give more) for weeks and months according to the circumstances of the case.

The Chairman did not know if it was possible to prevent well-disposed people from giving money without any enquiry into the applicants' circumstances. He believed the Charity Organisation Society had sent round notices asking people not to do mischief on this enormous scale.

A gentleman present, associated with the work of the Charity Organisation Society, said these notices had been sent on the occasion of an unusual amount of street begging cases for the police really. In reference to the work in Boston, there was a considerable difference, he thought, between the population, circumstances, and methods, followed in London and in that city; it would be hard to draw an analogy. Pauperism was not increasing here at home. The actual amount of very destitute persons was small in comparison with what it had been. The result of some recent enquiry in several districts of London had showed that the number of those belonging to the criminal population was comparatively trifling when set beside the number of those who were well-to-do, and were earning fairly good wages. There was certainly need of information and intercommunication in particular districts. He agreed with the views expressed as to the Mansion House Fund.

Mr. Crispin said the Bishop of Bedford had spoken strongly against indiscriminate charity; and the Bishop of Marlborough felt the injury of it so much that he even said those practising it deserved to be punished.

The Chairman thought Mr. Burdett hit the nail on the head in speaking against giving to people without knowing anything about them. Not alms, but a friend, he believed, was the Boston principle. People who gave half-a-crown in reply to a piteous tale would not give sixpence to an organisation which aimed at doing good systematically. How to get these people to do the right thing in the right way was what he wanted to know.

Mr. Stammer, a working man, said it was desirable especially to encourage thrift amongst the working people, who would value help in that way far more than charity in the winter time, and times of enforced idleness. Many of the half-crowns given as described would be better spent if the working-man was told that when he saved twenty shillings he would get 10 per cent. for it.

The Chairman said they must get all the workers in the district together to consider such a plan as Mr. Burdett had submitted. If he would show them how to give their sympathy, their enthusiasm, and perhaps their money, many would be glad to do it. As to Mr. Stammer's suggestion, he (the chairman) did not like the principle of giving large bonuses for money. As to technical schools, the Campden Trustees had established one for boys in St. Clement's parish, near this, and had set up flourishing evening classes for girls in the room they now occupied.

Mr. Burdett said Boston, as had been pointed out, was different from London. It had not the same old traditions to fight against in charitable works. For instance, there were free as well as pay beds in American hospitals, but they would find the free beds used by anyone but Americans. He found that great difficulty was to convince people that they ought to move at all, and that it was desirable to move in any direction but the one in which they had gone all their life. His proposal was not meant to interfere with existing agencies, but to bring all into harmony by an intelligent system. This plan, when printed, could be sent to all the workers, of all denominations, in the district, or a committee of the most active workers could be formed to take whatever steps seemed desirable. He naturally came first to his own parish, where they had an able organiser at the head of affairs. The details would be largely modified as they gained experience and knew what the requirements of their own district were. In Paddington they had been met by difficulties at first, but when they taught artisans to do spade work in planting Paddington recreation ground, then they said: "This is a practical method; how much money do you want?" Thrift and other things mentioned were details of the plan. As to giving money in the streets, they should resolve, "We want to free ourselves from those people." There had been a fall of about 14,000 in London paupers in the last ten years, but the relief given had risen more than three-quarters of a million. Every pauper in London cost £22 5s. 2d., while in the country only £10 12s. The loss in London was twice as heavy, and the present system did not cure it. They ought to be able to know that no one in their district was allowed to starve. That was the kernel of his proposal.

Mr. Crispin moved that Mr. Burdett be requested to print his proposals, with a view to having them brought under the consideration of all workers in All Saints' parish, who should be invited to a subsequent meeting to consider whether these proposals as a whole, or with modification, should be adopted in this parish.

Rev. C. J. Ward seconded this resolution, which was put and carried.

The Chairman said he hoped the printed paper would be sent amongst the laity as well as the clergy. Mr. Burdett was willing to give his library for a meeting of those willing to see the scheme carried out. If another public meeting was thought desirable Mr. Burdett would attend.

The meeting then separated.

